# Prognostic significance of dysregulation of shelterin complex and its correlation with telomere length and cytogenetics in multiple myeloma

**DOI:** 10.1186/s43141-023-00504-x

**Published:** 2023-05-03

**Authors:** Akanksha A. Kalal, Reshma A. Shetty, Akshay Bairapura Manjappa, Nagaraj V. Kulkarni, Prashanth Shetty

**Affiliations:** 1 KSHEMA Center for Genetic Services, KS Hegde Medical Academy, NITTE (Deemed to Be University), Mangaluru, Karnataka India; 2 Department of Anatomy, KS Hegde Medical Academy, NITTE (Deemed to Be University), Mangaluru, Karnataka India; 3 Chromosome and Plasmid segregation Lab, Department of Bioscience and Bioengineering, Indian Institute of Technology, Bombay, Maharashtra India

**Keywords:** Telomere-associated genes, Telomere length, Multiple myeloma, Overall survival, Cytogenetics

## Abstract

**Background:**

MM (multiple myeloma) is a bone marrow disease with the accumulation of malignant plasma cells characterized by the neoplastic transformation of differentiated B cells.

The onset and progression of cancer are greatly influenced by telomere dysfunction. We aimed to study the biomarker potential and prognostic significance of shelterin complex and hTERT. Telomere length and gene expression were measured using real-time quantitative reverse transcription-polymerase chain reaction (RT-qPCR), and these results were further correlated with clinical parameters.

**Results:**

Our study showed increased expression of all genes in complex, hTERT, and TL in MM (*n* = 72) in comparison with controls (*n* = 31). TRF2 (*P* = 0.025) and hTERT (*P* = 0.0002) displayed significant association among cytogenetic analysis. The receiver operative curve showed POT1 and RAP1 with a greater area under the curve (AUC). RAP1 (*P* = 0.020) and hTERT (*P* = 0.037) displayed to be independent prognostic markers for overall survival. Clinical parameters and genes were observed to be significantly correlated.

**Conclusion:**

Our study findings showed variation in telomere-associated genes and suggest the participation of these genes as prognostic markers in MM. These results all together highlight the evaluation and role of genes involved in telomeric alteration and TL, providing the opportunity to study new therapeutic approaches in patients with MM.

**Supplementary Information:**

The online version contains supplementary material available at 10.1186/s43141-023-00504-x.

## Background

Hematological cancer, multiple myeloma (MM) causes an accumulation of clonal plasma cells in the bone marrow, further characterized by secretion of monoclonal protein or M protein in urine or blood. MM progression is a dynamic and multi-step process involving cell differentiation, proliferation, and survival. MM proceeds from non-malignant plasma cells to malignant mutant clones by progressive genetic events resulting in immortal growth and neoplastic phenotype [1]. The transformation of the myeloma cell clone depends on genetic and epigenetic processes and the interaction between the BM microenvironment and malignant plasma cells [2]. Expansion of mutant cell clones may result from inhibition or induction of apoptosis, with a low rate of apoptosis in myeloma cells. The pathogenesis of MM is similar to in vitro immortalization and transformation of somatic cells into a cellular oncogene. Regulation of length of telomere and activation of telomerase is a critical pace for in vitro immortalization.

Telomeres are specialized DNA and protein structures comprised of tandem repeats of (TTAGGG) and telomere-related proteins at chromosome ends. Telomeres serve a dominant role in sustaining genome stability and protecting ends of the DNA from nucleolytic degradation, DNA fusion, recombination, and repair mechanism [3]. Telomeric DNA is shortened from the end replication problem, with division of each cell leading to tumorigenesis and telomere dysfunction with implications for oncogenesis. Telomerase regulates telomere length (TL); it is a ribonuclear protein complex with an RNA component (hTR) and catalytical reverse transcriptase protein (hTERT). Telomeric ends are successively augmented with additional repeats which compensates for telomeric reduction. The key regulators of telomerase activity are the shelterin complex and hTERT transcription [4].

Shelterin is a protein complex of six telomere-specific proteins, functioning as a safeguard and protecting the ends of the telomere. The core complex of shelterin is composed of telomere repeat binding factors 1 and 2 (TRF1, TRF2), protection of telomeres 1 (POT1), telomeric repeat binding factor 2 interacting protein (RAP1), TPP1 or adrenocortical dysplasia homolog (ACD), and TRF1 interacting nuclear factor 2 (TIN2) [5]. TRF1 and TRF2 are considered negative telomere length regulators where they bind to double-stranded DNA through their myb domain. TRF1 allows telomere shortening by inhibiting telomerase activity. TRF2 is involved in t-loop formation, protecting, and capping 3′ single-stranded overhang. POT1 is essential for preserving telomeric DNA and safeguarding chromosomes 5′ ends. POT1 has high specificity for single-stranded DNA overhangs promoting suppression of ATR pathways. RAP1 associates with TRF2 to bind to DNA, and its role is in inhibiting non-homologous end joining of telomeres [6]. TPP1 acts as a positive regulator in maintaining TL. TIN2 recruits TRF1, TRF2, TPP1, and ACD to telomere and acts as a scaffolding protein.

Very few studies have reported on the telomere and its functions, and the information present with respect to the Indian population is very scarce. The current study aimed to investigate the mRNA expression of shelterin complex in correlation with hTERT expression and TL with cytogenetic results and clinical parameters of MM subjects.

## Methods

### Patients and study sample

Seventy-two MM patients recruited at the Department of Oncology, KS Hegde Charitable Hospital, Karnataka, India, were enrolled in the study. For cytogenetic analysis, bone marrow in the heparin vacutainer and peripheral blood in the EDTA vacutainer was collected for expression study. The Institutional ethics committee approved the study, and all the samples were collected after the patients’ written informed consent. The patients’ clinical parameters were taken from clinical records.

### Conventional cytogenetics

The cytogenetic analysis was performed on all the collected samples. Cell cultures of bone marrow were incubated for 24–48 °C in a CO_2_ incubator. Culture was treated with colcemid (0.08 μg/mL, Gibco) and harvested and fixed using Carnoy’s fixative (methanol; acetic acid 3:1). The metaphase chromosomes were banded by Giemsa stain and analyzed using GENASIS software (Applied Spectral Imaging, Edingen - Neckarhausen, Germany).

### Quantification of relative mRNA expression of genes and relative telomere length by real-time polymerase chain reaction (RT-qPCR)

#### Isolation of RNA

Total RNA was extracted from whole blood by the phenol–chloroform method using RNA Iso plus reagent (Takara, Japan). 0.5 μg of RNA was transcribed to cDNA using Prime Script RT reagent kit (Takara, Japan), with a total volume of 20 μl cDNA.

#### Isolation of DNA

Genomic DNA (gDNA) was extracted from the blood drawn in the EDTA tube. The blood sample was centrifuged with RBC lysis solution (NH_4_Cl, KHCO_3_, Na_2_. EDTA). The residual RBC lysate was suspended with a cell lysing solution (50 mM Tris HCl, 50 mM EDTA, 10 mM NaCl, 1% SDS) and further digested overnight, followed by a protein precipitating solution (Qiagen). The lysate was centrifuged, and the supernatant was collected in 2% isopropanol and centrifuged again. The pellet consisting of DNA was washed with 70% ethanol, and the DNA was allowed to precipitate. The DNA pellet was air-dried at room temperature for 5 min, and gDNA was resuspended in 50 μl of nuclease-free water [7].

#### Relative mRNA expression

The RT-qPCR assay was performed using TB Green Premix Ex Taq II (Takara, Japan), with a final volume of 10 μl of PCR reaction. As a template, 1 μl of cDNA was taken. Each reaction was carried out in triplicates. The cyclic conditions were as follows: 1 cycle at 95 °C for 10 min, 94 °C for 30 s, 40 cycles at 60 °C for 1 min, and 95 °C for 30 s, and 65 °C for 30 s and 95 °C for 30 s. The mRNA expression of TRF1, RAP1, TRF2, TIN2, POT1, TPP1, hTERT, and Glyceraldehyde-3-Phosphate dehydrogenase (GAPDH) was used to normalize gene expression. For TL analysis, the primers used are presented in Table 1, and HBB (Human beta-globin) was used as an endogenous reference gene for the estimation of TL. 2^−ΔΔCt^ method was used to calculate and evaluate relative mRNA expression [8]. The primers used were previously described by Moazzam et al. [9]. The sequences of the genes are given in Table 1. Spectral data were captured and analyzed using Agilent Aria 1.8 (California).

**Table 1:** The primer sequences of the genes

Genes	Primer sequence
TRF1	**F:** GTACCCAAGCGAGCCATTTA
	**R:** GAAACGACGAGGAGCAGTC
TRF2	**F:** CTTGGGTGGAAGAGGATGAA
	**R:** TGACCCACTCGCTTTCTTCT
POT1	**F:** GCTCTGGCTTTGCATCTTTG
	**R:** GGTGCCATCCCATACCTTTAG
TIN2	**F:** CTGAGCCCATGGAACAGAAT
	**R:** GGTGAGCCGAGATTCCTAAAG
ACD (or TPP1)	**F:** GTCCCAGCTTCTGGATGAAA
	**R:** AGGCTATGAGGGTCAGAGATAG
RAP1	**F:** TAACGCCTTGTGGAAAGCGA
	**R:** CAGACGCTAAGAAGGCGGAA
hTERT	**F:** CTACTCCTCAGGCGACAAGG
	**R:** TGGAACCCAGAAAGATGGTC
GAPDH	**F:** ATGTTCGTCATGGGTGTGAA
	**R:** GTCTTCTGGGTGGCAGTGAT
TELO GC	**F:** ACACTAAGGTTTGGGTTTGGGTTTGGGTTTGGGTTAGTGT
	**R:** TGTTAGGTATCCCTATCCCTATCCCTATCCCTATCCCTAACA
HBB	**F:** GCTTCTGACACAACTGTGTTCACTAGC
	**R:** CACCAACTTCATCCACGTTCACC

## Statistical evaluation

GraphPad Prism (version 5; Software GraphPad, USA) was used for statistical analysis. Continuous data were summarized as median (range) and categorical data as percentage and mean ± SD. Mann-Whitney and Student *t*-tests were used to compare two groups, while one-way ANOVA for multiple group comparison. The receiver operative curve (ROC) was used to determine each gene’s expression cut-off value. The log-rank test was performed to compare the estimated overall survival using the Kaplan-Meier curve. Spearman rank correlation was done to determine the correlation between the gene expression, TL, and clinical parameters. *P* < 0.05 is considered to be statistically significant.

## Results

Seventy-two MM patients were recruited for the study. The clinical and laboratory characteristics of MM cases are given in Table 2. Out of 72 MM patients, conventional cytogenetics (CC) revealed normal karyotype in 56% (40/72) and abnormal karyotype in 39% (28/72) (Supplementary Table 1) and 5% (4/72) with culture failure.

**Table 2:** Clinical and Laboratory parameters

Characteristics	Percentage/median (range)
Age (years)^a^	61.5 (42–75)
Male (%)^b^	42 (58%)
Female (%)^b^	30 (42%)
**ISS staging**	
I/II/III/undetermined	18/26/23/5
IgG/IgA/undetermined	55/12/5
Light chain type, kappa/lambda/undetermined	42/25/5
**Plasma cells in bone marrow**	
< 10%	13
10–25%	21
> 25%	38
Hb (g/dL)^a^	9.5 (4.9–15)
ESR (mm/h)^a^	59.5 (3––150)
Total protein (g/dL)^a^	7.75 (3.44–16.7)
Albumin (g/dL)^a^	3.52 (1.1–5.2)
Globulin (g/dL)^a^	3.6 (1.43–14.43)
Bilirubin direct (mg/dL)^a^	0.13 (0.04–0.9)
Bilirubin indirect (mg/dL)^a^	0.28 (0.07–5.43)
Bilirubin total (mg/dL)^a^	0.51 (0.1–5.63)
SGOT (U/L)^a^	22.35 (2–65)
SGPT (U/L)^a^	19 (5–68)
ALP (U/L)^a^	94 (31–1136)
Calcium (mg/dL)^a^	9.05 (5.4–16.8)
Sodium (mEq/L)^a^	135 (124–142)
Potassium (mEq/L)^a^	3.4 (2.74–40.4)
Blood urea (mg/dL)^a^	31.25 (6.3–140.8)
Uric acid (mg/dL)^a^	5.75 (1.85–21.6)
Creatinine (mg/dL)^a^	1.1 (0.44–6.99)
**Cytogenetic analysis**	
Normal karyotype	40 (56%)
Abnormal karyotype	28 (39%)
Culture failure	4 (5%)

Values presented as ^a^median (range) and ^b^percentage

### mRNA expression

**Fig. 1:**
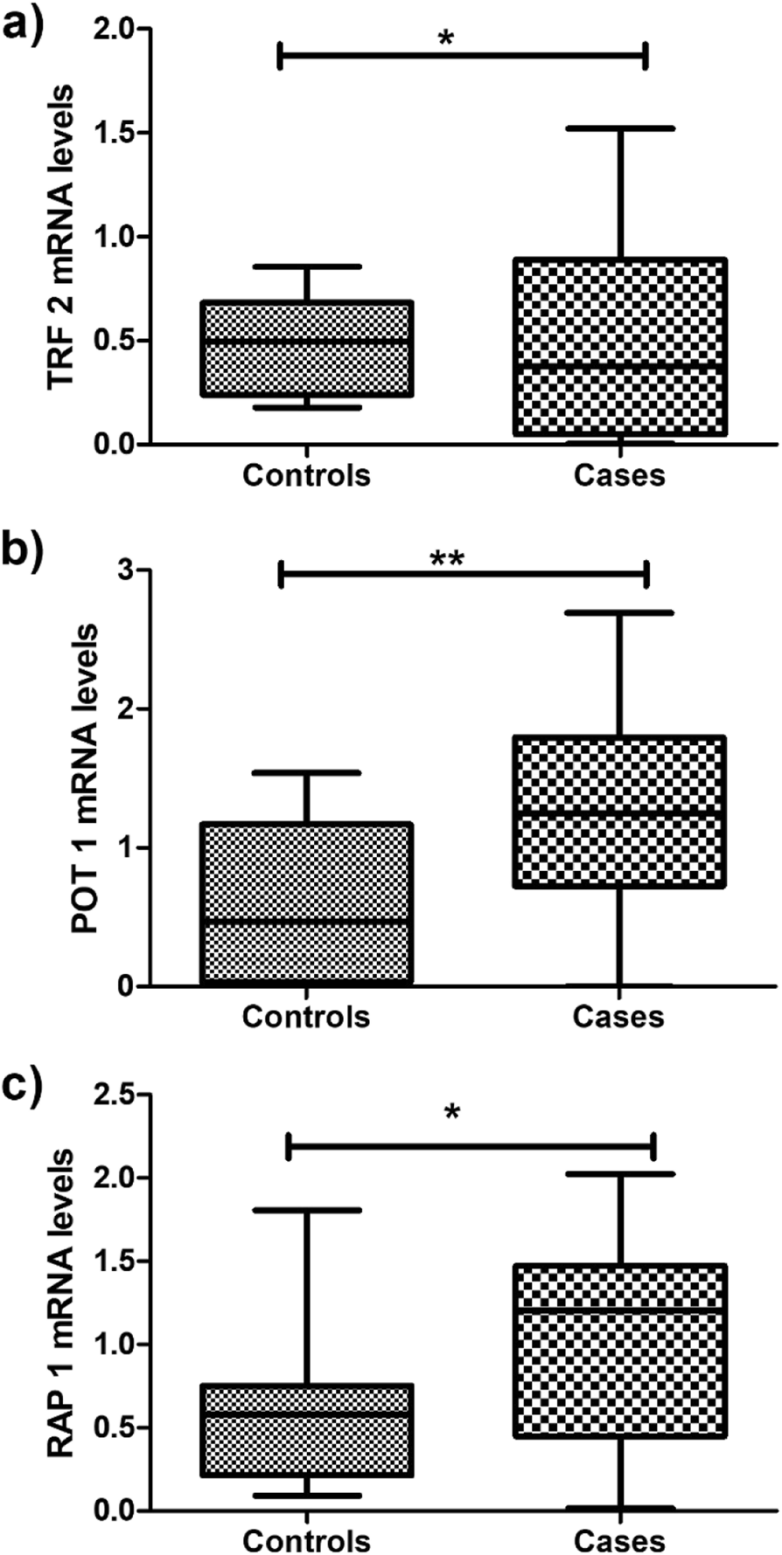
Box and whisker plot exhibiting relative mRNA expression of **a** TRF2, **b** POT1, and **c** RAP1

The mRNA expression of shelterin complex genes and hTERT was evaluated in 72 MM cases and 31 controls. mRNA expression of TRF2 (*P* = 0.0188), POT1 (*P* = 0.0047), and RAP1 (*P* = 0.045) displayed a significantly higher expression in MM cases than controls (Fig. 1). TRF1 (*P* = 0.146), TIN2 (*P* = 0.3931), ACD (*P* = 0.2847), and hTERT (*P* = 0.4152) showed non-significant higher expression in cases than controls.

**Fig. 2﻿:**
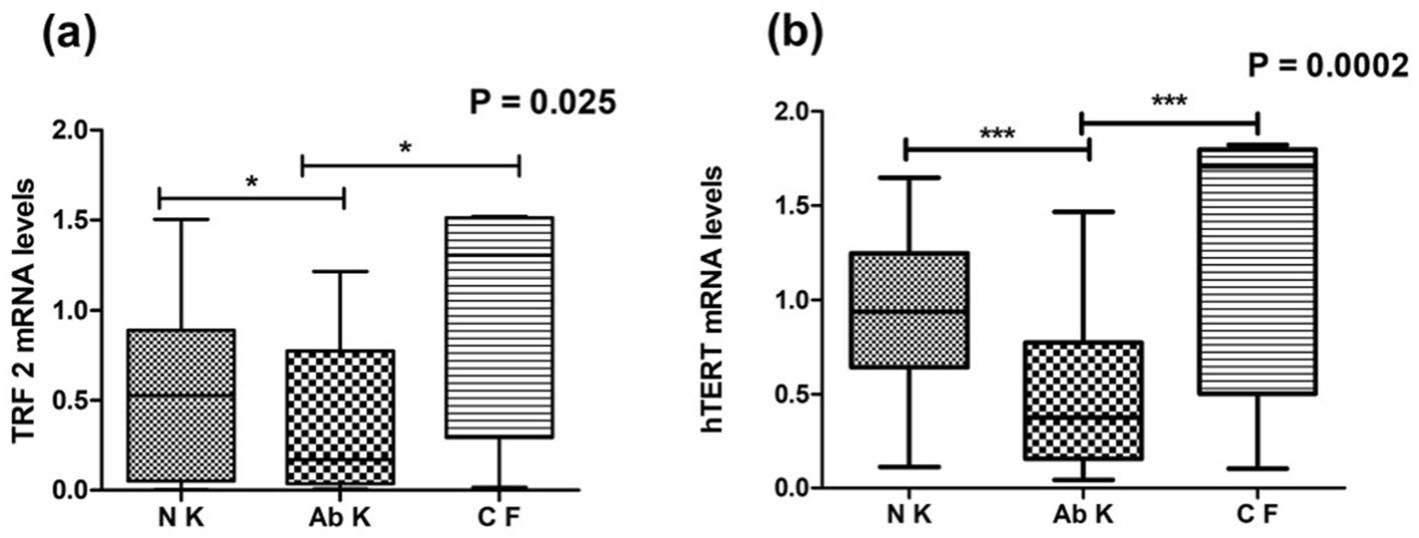
Box and whisker plot showing relative mRNA expression of **a** TRF2 among NK, Ab K, and CF and **b** hTERT among NK, Ab K, and CF. NK, normal karyotype; Ab K, abnormal karyotype; CF, culture failure

No statistical significance between the group based on cytogenetic analysis for the genes TRF1, POT1, RAP1, TIN2, and ACD was observed. TRF2 (*P* = 0.025) and hTERT (0.0002) displayed a significant difference between normal karyotype, abnormal karyotype, and culture failure (Table 3) (Fig. 2). Among abnormal karyotypes, no statistical difference was observed for all the shelterin complex genes and hTERT (Table 4).

**Table 3:** Gene expression in association with cytogenetic analysis

Cytogenetic analysis	*P* value						
	**TRF1**	**TRF2**	**POT1**	**RAP1**	**TIN2**	**ACD**	**hTERT**
Normal karyotype	0.978	0.025*	0.980	0.615	0.452	0.339	0.0002***
Abnormal karyotype							
Culture failure							

^*^P < 0.05, ^***^P < 0.001

**Table 4:** Gene expression among abnormalities

Abnormal karyotypes	*P* value						
	**TRF1**	**TRF2**	**POT1**	**RAP1**	**TIN2**	**ACD**	**hTERT**
Hyperdiploidy	0.849	0.620	0.526	0.467	0.248	0.460	0.199
Hypodiploidy							

The relative TL displayed no significant difference between cases and controls. Non-significant expression was observed, with expression higher in cases than in control (*P* = 0.421) (Supplementary Table 2 and Supplementary Fig. 1).

**Fig. 3:**
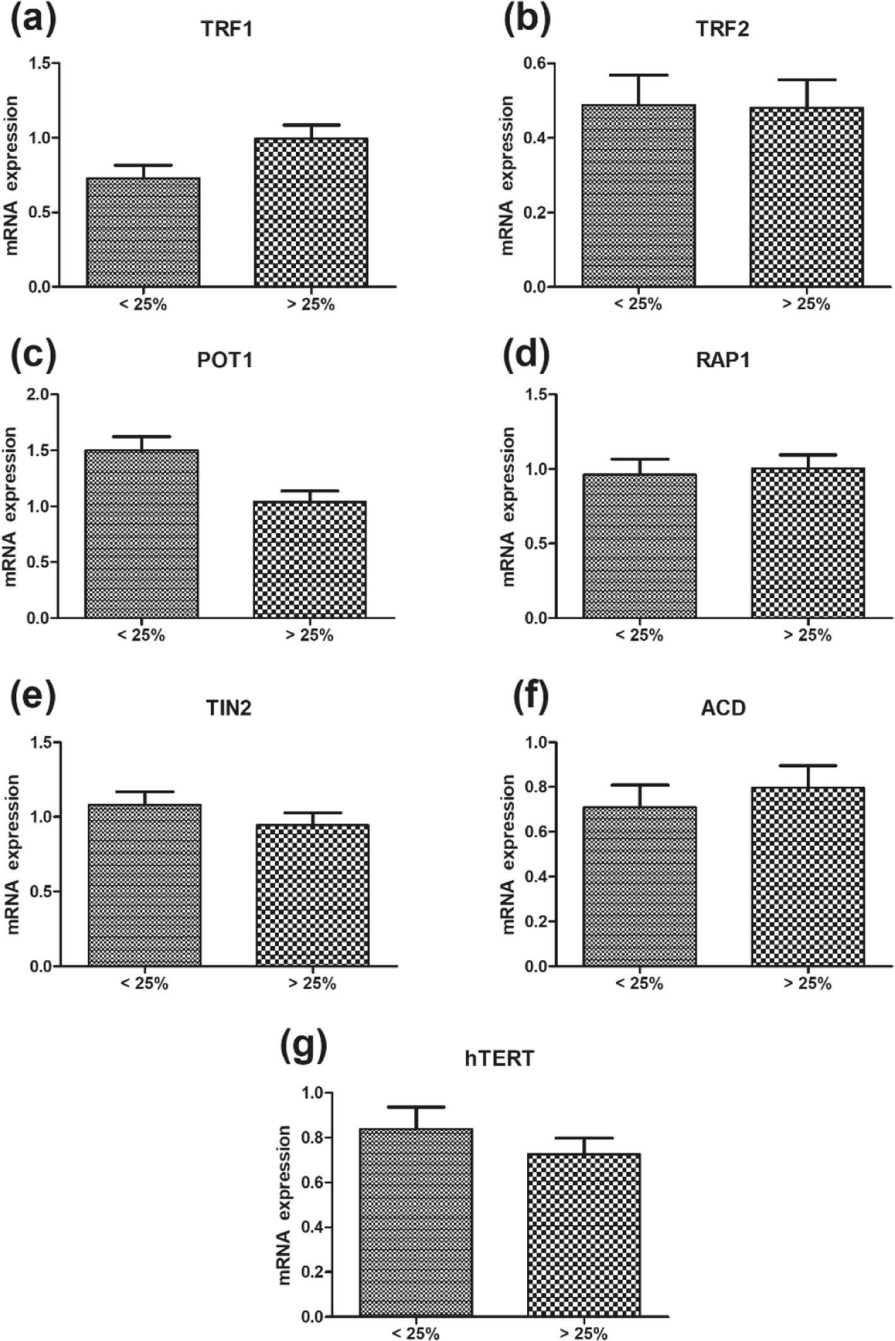
Column bar graph representing mRNA gene expression in MM cases with < 25% PC and ≥ 25% PC

The mRNA expression of TRF1, RAP1, and ACD genes with respect to PC% showed increased expression in patients with PC% ≥ 25%, and TRF2, POT1, TIN2, and hTERT displayed higher expression in patients with PC% < 25% (Fig. 3).

### hTERT, shelterin complex, and mTL’s predictive value in MM

**Fig. 4:**
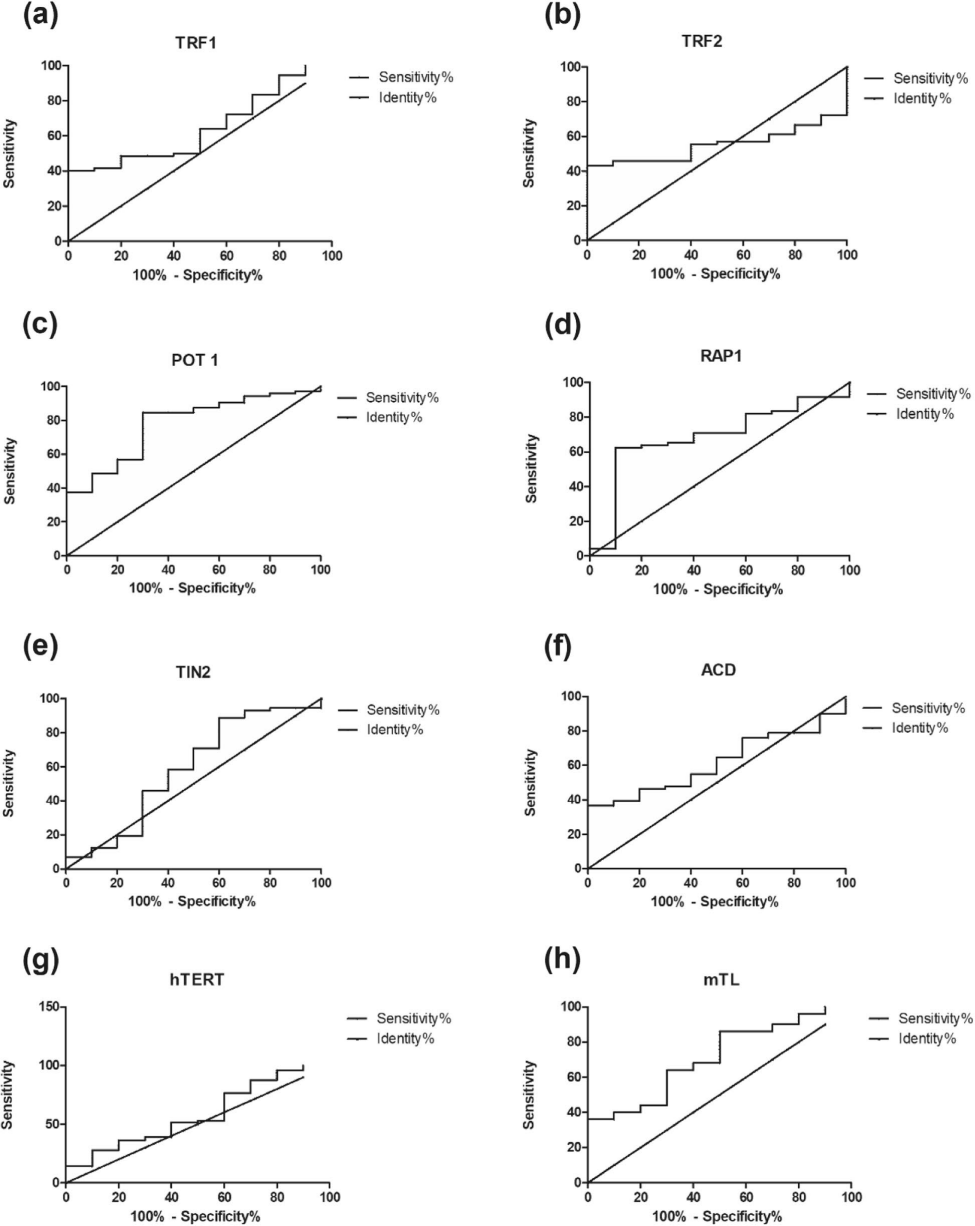
Receiver operative curve (ROC) and area under the curve (AUC) for **a** TRF1, **b** TRF2, **c** POT1, **d** RAP1, **e** TIN2, **f** ACD, **g** hTERT, and **h** mTL

Receiver operator curve (ROC) was generated to assess the degree of each parameter’s association to the likelihood of contracting a disease (Fig. 4). Out of all genes analyzed, POT1 (AUC = 0.7778) and RAP1 (AUC = 0.6861) had greater area under the curve (AUC). (Supplementary Table 3). Patients were distributed based on the cut-offs obtained by ROC curves. These cut-offs were used to stratify and study the overall survival based on the patient’s gene expression.

**Fig. 5:**
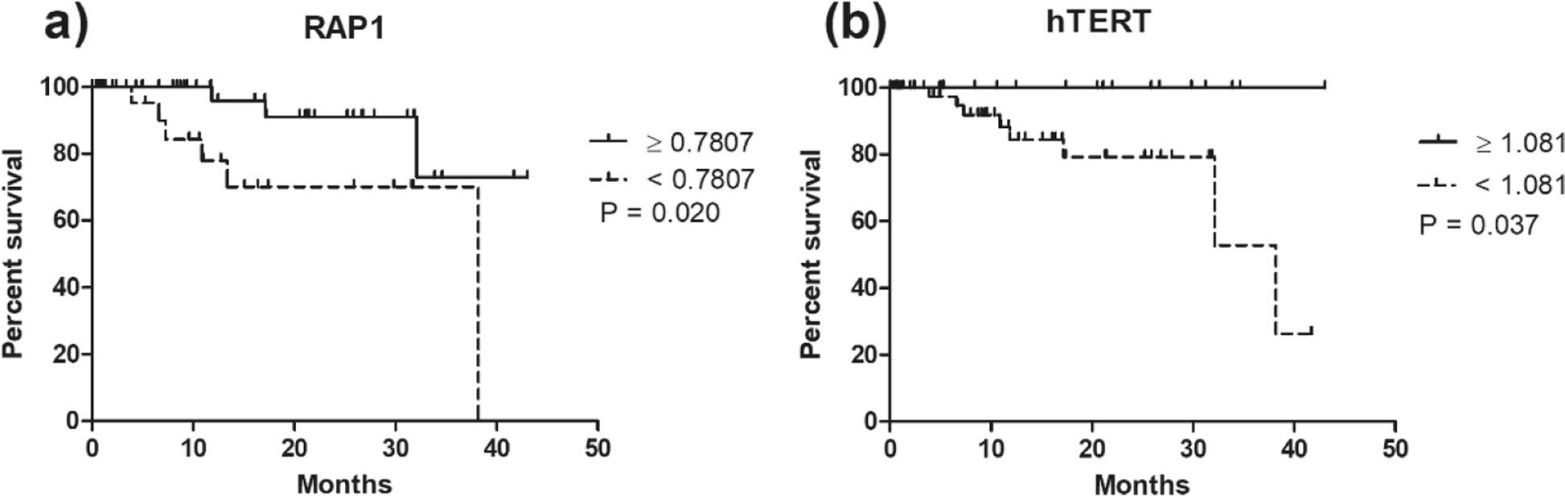
Kaplan–Meier curve showing overall survival for MM subjects distinguished by **a** RAP1 (*P* = 0.020) and **b** hTERT (*P* = 0.037)

Kaplan–Meier estimates in MM patients of overall survival displayed significant independent prognostic factor for RAP1 (*P* = 0.020) and hTERT (*P* = 0.037). A significant difference between low and high mRNA levels of RAP1 and hTERT was seen (Fig. 5). The mean OS was 12.2 vs 15.8 months with a hazard ratio of 0.185 (95% CI −0.044 to 0.769) for RAP1 and 13.2 vs 17.8 months with a hazard ratio of 4.78 (95% CI −1.099 to 20.80) for hTERT. No significant difference for Kaplan-Meier estimates for other genes along with mTL was observed (Supplementary Table 4 and Supplementary Fig. 2).

### Clinicopathological parameters

**Fig. 6:**
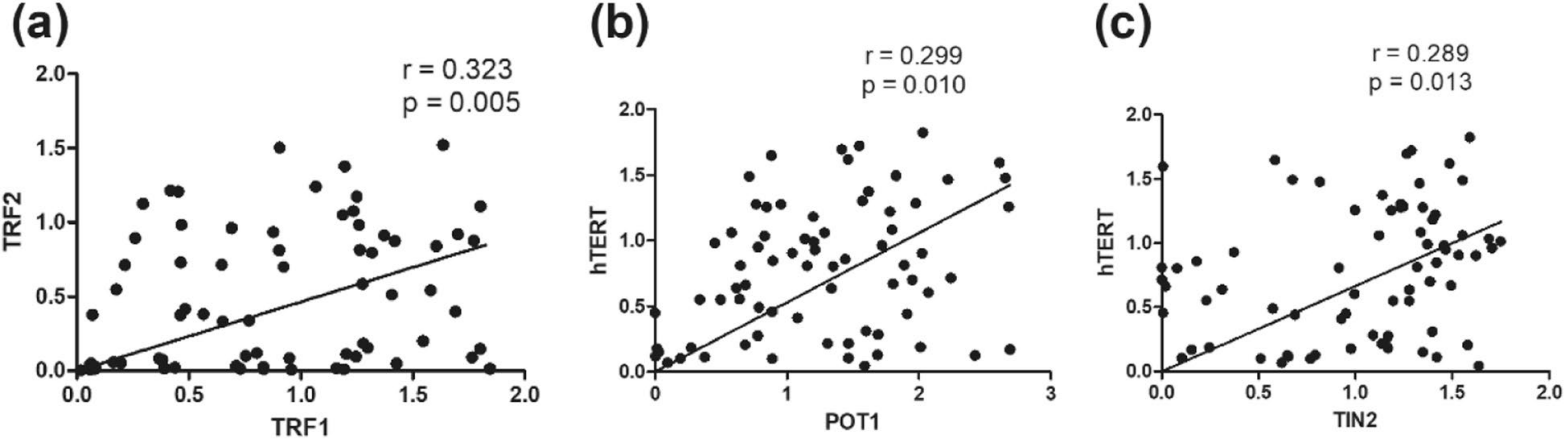
Graphical representation of significance and correlation level between **a** TRF2–TRF1, **b** hTERT–POT1, and **c** hTERT–TIN2

**Fig. 7:**
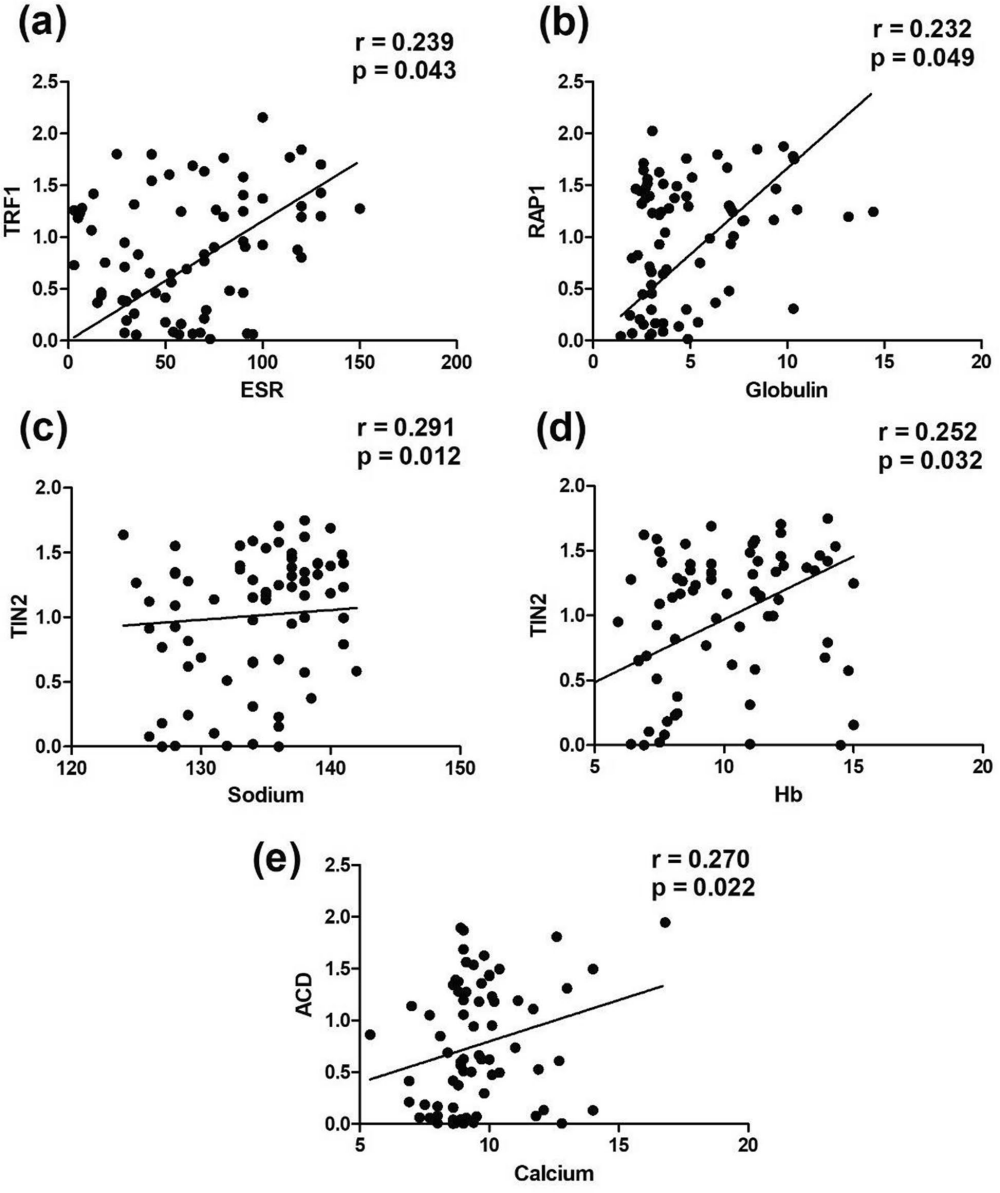
Graphical illustration of strength of correlation and significance between **a** TRF1–ESR, **b** RAP1–Globulin, **c** TIN2–Sodium, **d** TIN2–Hb, **e** ACD–Calcium

**Fig. 8:**
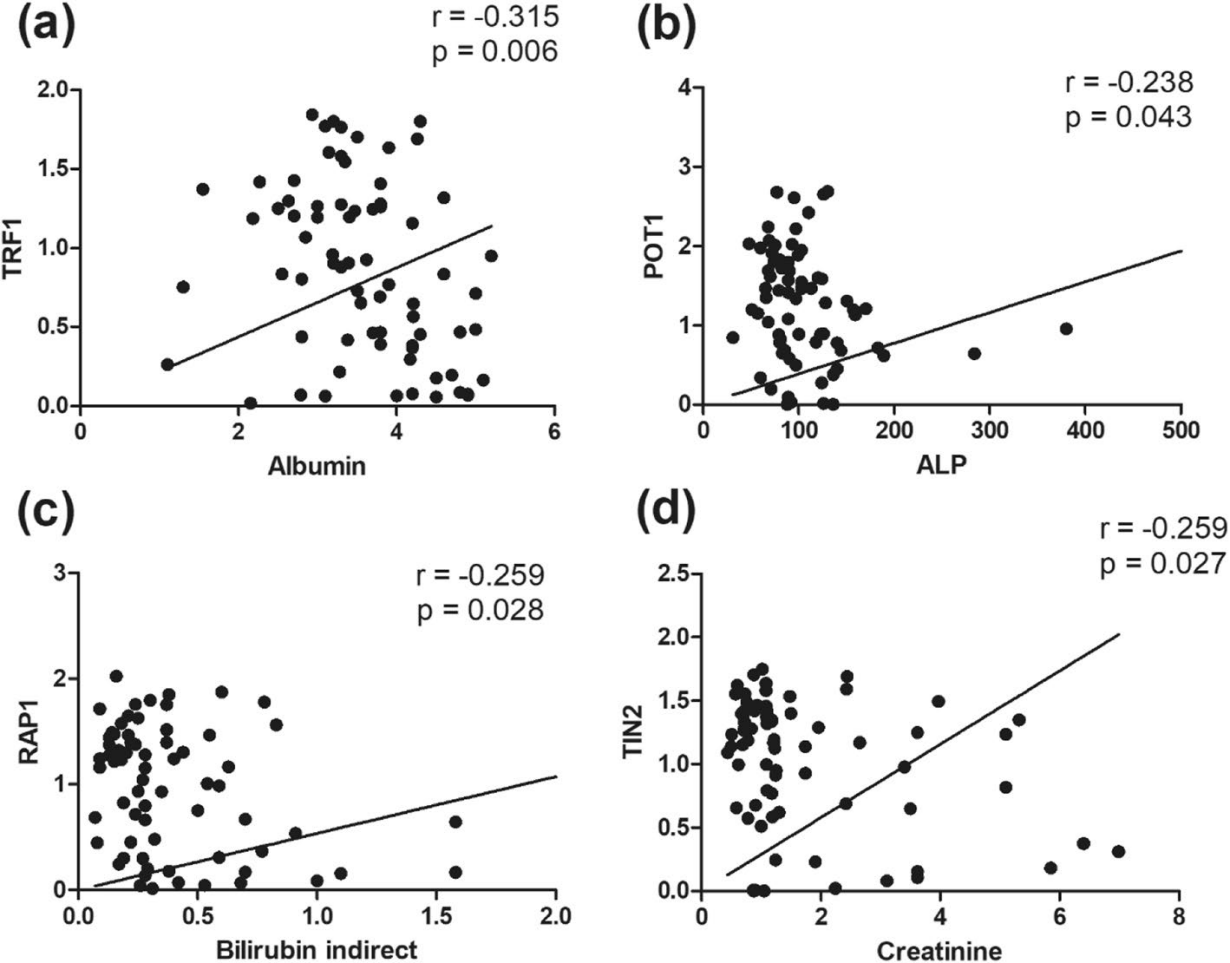
Graphical depiction of correlation strength and significance level between **a** TRF1–Albumin, **b** POT1–ALP, **c** RAP1–Bilirubin indirect, **d** TIN2–Creatinine

All of the mRNA levels for every gene were analyzed for association with clinical and laboratory parameters. TRF1–TRF2, POT1–hTERT, and TIN2–hTERT positively correlated with “r” as 0.32, 0.29, and 0.28 respectively (Fig. 6). TRF1–ESR, Globulin–RAP1, Sodium–TIN2, Hb–TIN2, and Calcium–ACD showed a positive correlation with “r” as 0.23, 0.23, 0.29, 0.25 and 0.27 respectively (Fig. 7). Albumin–TRF1, ALP–POT1, Bilirubin indirect–RAP1, and Creatinine–TIN2 showed a significant negative correlation with “r” as −0.31, −0.23, −0.25, and −0.25, respectively (Fig. 8) (Supplementary Tables 5 and 6).

## Discussion

MM is a composite genetic condition with proliferative malignant plasma cells in the bone marrow. In human cancers, telomeres are thought to play important roles, and researchers have looked into the TL role and activity of telomerase in cancer development. In the present study, we tend to determine the mRNA levels of telomeric proteins and TL in MM and healthy individuals. It is the only study from South India describing the participation and modification of these proteins in tumorigenesis.

Human telomeres require the shelterin complex and its related components to operate correctly and be maintained. Most malignancies, including MM, have changed these molecules in conjunction with human telomerase [10]. Our study showed significantly elevated mRNA expression of all shelterin complex genes in MM subjects. Previous studies have shown higher mRNA expression of these genes in human gastric carcinomas, hepatocarcinogenesis, and adult cell leukemia [11–13]. In our study, downregulation of TRF1 was seen, which was consistent with previously published data. TRF1 is a negative regulator of TL, which limits elongation, resulting in stable TL. Long telomeres block the telomerase-mediated elongation by recruiting a large amount of TRF1 proteins [14]. HT1080, a telomerase-positive cell line, has shown progressive shortening of telomere with overexpression of TRF1, suggesting its repressor role [15]. Several studies showed the downregulation of TRF1 [16–18] while the others showed upregulation [12, 19–21]. This data may account due to different tumor stages and types. High TRF2 levels have been shown to delay the senescence of short telomeres, therefore the high expression of TRF2 [22].

Significant higher expression of POT1 and RAP1 was observed in our study. Among the shelterin complex, POT1 is the most effective gene with excessive specificity for ssDNA of the telomere. Over-expression of POT1 with unfavorable prognostic factors and poor clinical outcomes were observed in MM compared to MGUS. An increase in expression of POT1, RAP1, TIN2, and ACD has been observed in MM cases than monoclonal gammopathy of undetermined significance (MGUS), predicting telomere role and as prognostic markers [23]. POT1 expression is highly influenced by disease stage and TL. POT1 expression decreased as TL was shortened in gastric carcinoma by Kondo et al. [20]. RAP1 regulates TL negatively, which is recruited to telomere by TRF2 [24].

In our study, the hTERT gene analysis revealed a higher mRNA expression explaining high telomerase activity in MM patients. A study by Panero et al. shows expression of hTERT was higher with shortened TL in MGUS patients suggesting its role in the progression of MGUS to MM [4]. To evade the shortening of telomeres, tumor cells increase telomerase activity, increasing the expression of hTERT. Klapper et al.'s study showed the upregulation of activity of telomerase as a result of transcriptional upregulation of hTERT in Burkitt’s lymphoma [19]. In the early stage of stomach carcinogenesis, hTERT expression may be considered one of the preconditions for the activation of telomerase [25]. Further, mRNA expression of TRF1, RAP1, and ACD were associated with increased PC% suggesting its significant expression with disease severity.

ROC curves were plotted and classified each gene into two groups based on the cut-offs obtained. POT1 and RAP1 displayed significant area under the curve, showing their potential as biomarkers for better disease prediction. A significant association was observed between OS and RAP1 expression and OS with hTERT expression. These results were consistent with RAP1 in yeast [26].

We observed a significant association of TRF2 and hTERT genes among cytogenetic subgroups. One putative mechanism, such as telomere dysfunction, may lead to clinical and genetic heterogeneity in MM. Dysfunctioning short telomeres are susceptible to DNA repair activity, resulting in chromosome fusion driving clonal evolution and genomic instability [27–29].

TL, which is essential for telomere dysfunction and cell survival, was also studied. In our study, cases showed non-significant higher expression than controls and no association between overall survival and TL. Telomeres are involved in the process like chromosomal instability, which can be used as a risk marker in the development of MM [30]. mRNA expression of TRF1–TRF2, POT1–hTERT and TIN2–hTERT displayed a significant positive correlation. mRNA levels of TRF1 correlated positively with ESR, whereas TIN2 mRNA levels significantly correlated with hemoglobulin and sodium.

ESR being a marker of inflammation significantly correlated with mRNA expression of TRF1 revealing inflammation and progression of disease. Renal dysfunction and hypercalcemia are the most common features of MM, therefore displaying a significant correlation of calcium, with mRNA expression of shelterin.

A significant negative correlation was observed between Albumin–TRF1, ALP–POT1, bilirubin indirect–RAP1, and Creatinine–TIN2. Hypoalbuminemia is a common finding and has close association with clinical stage of the disease and tumor burden [31]. In our study, a significant positive correlation between globulin and RAP1 and a negative correlation between TRF1 and albumin were observed. Increase in globulin levels and decrease in levels of albumin reflect increases in M proteins in MM. These results provide evidence of these genes’ involvement in the regulation of telomeres and progression of MM.

## Conclusion

In conclusion, the present work indicates a global alteration of shelterin complex genes and hTERT and provides insight into TL involvement in the progression of MM. TRF2, POT1, and RAP1 displayed significant mRNA expression in MM patients. POT1 and RAP1 were also associated with overall survival and correlated significantly with clinical parameters. For immortalizing cells, TL regulation and maintaining the end structure of telomere by its related proteins are essential. In our study, we can reflect the importance of TRF2, POT1, RAP1, and hTERT influencing the efficiency as prognostic markers besides TL and telomerase activity.

Therefore, these results contribute to the comprehensive role of telomere-associated genes in telomere dysfunction in MM. Our study revealed the expression status of telomere-associated genes and mRNA expression status varies due to tumor heterogeneity and therefore can be used as a potential target for establishing a therapy strategy in MM. Further studies on large cohorts may provide better results to validate their potential as biomarkers in MM.

## Supplementary Information

**Additional file 1: Supplementary Table 1.** Chromosomal abnormalities of the MM patients. **Supplementary Table 2.** Representation of mRNA expression levels of Shelterin complex genes, hTERT and mTL. Data represented in median (range). **Supplementary Table 3.** Representation of AUC (Area under Curve) obtained by Receiver operative curves. **Supplementary Table 4.** Kaplan- Meier analysis of all the genes. **Supplementary Table 5.** Representation of ‘r’ and ‘p’ values by spearman correlation between mRNA levels of Shelterin complex genes, hTERT and mTL. **Supplementary Table 6.** Representation of ‘r’ and ‘p’ values by spearman correlation between mRNA levels of Shelterin complex genes, hTERT, mTL and clinical parameters. **Supplementary Figure 1.** Box Whisker plot showing relative mRNA expression of A)TRF1, B)TIN2, C)ACD, D)hTERT and E) mTL. **Supplementary Figure 2.** Kaplan Meier curve showing overall survival for MM patients stratified by A) TRF1 (*P* = 0.873), B) TRF2 (*P* = 0.929), C) POT1 (*P* = 0.943), D) TIN2 (*P* = 0.421), E) ACD (*P* = 0.908) and F) mTL (*P* = 0.304).

## Data Availability

Not applicable.

## Abbreviations

MM: Multiple myeloma
TL: Telomere length
TRF 1: Telomere repeat binding factors 1
TRF 2: Telomere repeat binding factors 2
POT 1: Protection of telomeres 1
RAP 1: Telomeric repeat binding factor 2 interacting protein
ACD: Adrenocortical dysplasia homolog
TIN2: TRF1 interacting nuclear factor 2
EDTA: Ethylene Diamine Tetra Acetic acid
RT-qPCR: Reverse transcriptase quantitative polymerase chain reaction
gDNA: Genomic DNA
GAPDH: Glyceraldehyde-3-Phosphate dehydrogenase
ROC: Receiver operative curve
AUC: Area under the curve
CCs: Conventional cytogenetics
