# Supplementary material for: Prognostic significance of dysregulation of shelterin complex and its correlation with telomere length and cytogenetics in multiple myeloma
Source: J Genet Eng Biotechnol. 2023 May 3;21:50. doi: 10.1186/s43141-023-00504-x (PMC10154441; doi:10.1186/s43141-023-00504-x)

**Supplementary data:**

Supplementary Table 1: Chromosomal abnormalities of the MM patients.

| **Chromosomal**  **Abnormality** | **Karyotypes** |
| --- | --- |
| Hyperdiploidy | 55~108, XY* |
|  | 47, XY,+ Mar |
|  | 33~57,XX,-X,+3,+5,+7,+8,+11,+13,+ 5,+17,+19,+mar[cp26] |
|  | 50~52,XY,inv(1p),dup(3q),+i(7q),+9,+11,+15,+19,+20,+mar,+mar[cp15] |
|  | 51~53,XY,dup(1)(q21q31),+4,+5,+6,+8,+9,+11,+12,+13,+17,+18,+mar,+mar |
|  | 46~53,XY,+5,+6,+8,+15,+19,-20,+19,-21,+22,+mar,+mar,+mar,+mar |
|  | 55,XX,+5,+7,+8,+14,+15,+15,+19,+mar,+mar |
| Hypodiploidy | 41~45,XY,-6,-13,-14,-19,-20  42~45,XX,-10,-14,-18,-22  32~45,X,-2,-5,-9,-10,-11,-12,-13,-14,-16,-17,-19,-20,-21,-Y  43~45,XY,-9,-12,-15,-16,-22  40~45,X,-16,-19,-X  41~45,X,-12,-19,-21,-Y  40~45,X,-9,-11,-13,-15,-16,-17,-19,-22,-Y  40~45,X,-5,-18,-Y  41~45,XX,-1,-9,-10,-18,-19  41~45,XX,-6,-7,-17,-18,-20  40~45,XX,-3,-6,-9,-13,-15,-16,-17,-20,-21,-22  44~45,X,-9,-16,-20,-22,-Y  42~45,XX,-5,-13  44~46,XY,-12,-21  45~44, XY,-20,-21  42~45,XX,-9  44~45,X,-8,-X  44~45,X,-4,-12,-Y  41~45,XX,-10,-12,-18,-19,-21 |
|  | 34~45,XX,-4,-8,-12,-21 |
| Structural abnormalities | 46,XX,del(16)(q12q22) |
| * Counted, due to poor morphology of chromosomes and clumped nature could not construct the karyogram | |

Supplementary Table 2: Representation of mRNA expression levels of Shelterin complex genes, hTERT and mTL. Data represented in median (range).

|  | Controls | Cases |
| --- | --- | --- |
| TRF1 | 0.609 (0.013 – 1.519) | 0.868 (0.018 – 1.962) |
| TRF2 | 0.473 (0.134 – 0.855) | 0.484 (0.003 – 1.846) |
| POT1 | 0.583 (0.003 – 1.723) | 1.254 (0.001 – 2.815) |
| RAP1 | 0.593 (0.089 – 1.808) | 0.984 (0.014 – 2.294) |
| TIN2 | 0.778 (0.009 - 1.980) | 1.008 (0.0004 – 1.946) |
| TPP1 | 0.491 (0.032 - 1.122) | 0.765 (0.003 – 1.947) |
| hTERT | 0.574 (-0.81 – 1.767) | 0.779 (0.022 – 1.968) |
| mTL | 0.480 (0.053 – 0.901) | 0.794 (0.165 – 1.918) |

Supplementary Table 3: Representation of AUC (Area under Curve) obtained by Receiver operative curves.

| Parameters | Area under Curve (AUC) |
| --- | --- |
| TRF1 | 0.6431 |
| TRF2 | 0.5500 |
| POT1 | 0.7778 |
| RAP1 | 0.6861 |
| TIN2 | 0.5874 |
| ACD | 0.6164 |
| hTERT | 0.5806 |
| mTL | 0.7100 |

Supplementary table 4: Kaplan- Meier analysis of all the genes.

| **Genes** | **Months (mean low Vs high)** | **P value** |
| --- | --- | --- |
| TRF1 | 13.8 Vs 15.4 | 0.873 |
| TRF2 | 16.5 Vs 12.9 | 0.929 |
| POT1 | 15.5 Vs 14.2 | 0.943 |
| RAP1 | 12.29 Vs 15.8 | **0.020*** |
| TIN2 | 10.2 Vs 15.0 | 0.421 |
| ACD | 14.1 Vs 14.6 | 0.908 |
| hTERT | 13.2 Vs 17.8 | **0.037*** |
| mTL | 17.06 Vs 17.4 | 0.304 |

Supplementary table 5: Representation of ‘r’ and ‘p’ values by spearman correlation between mRNA levels of Shelterin complex genes, hTERT and mTL.

| **r** | **TRF1** | **TRF2** | **POT1** | **RAP1** | **TIN2** | **ACD** | **hTERT** | **mTL** |
| --- | --- | --- | --- | --- | --- | --- | --- | --- |
| **p** |  |  |  |  |  |  |  |  |
| TRF1 |  | 0.323 | 0.117 | 0.099 | 0.055 | 0.103 | 0.002 | -0.004 |
|  |  | **0.005*** | 0.327 | 0.406 | 0.641 | 0.390 | 0.982 | 0.974 |
| TRF2 |  |  | 0.146 | -0.072 | 0.055 | 0.130 | 0.063 | -0.086 |
|  |  |  | 0.218 | 0.542 | 0.641 | 0.277 | 0.595 | 0.550 |
| POT1 |  |  |  | 0.111 | -0.015 | 0.189 | 0.299 | -0.222 |
|  |  |  |  | 0.350 | 0.895 | 0.113 | **0.015** | 0.121 |
| RAP1 |  |  |  |  | 0.044 | 0.135 | 0.162 | 0.007 |
|  |  |  |  |  | 0.709 | 0.259 | 0.171 | 0.958 |
| TIN2 |  |  |  |  |  | 0.200 | 0.289 | -0.096 |
|  |  |  |  |  |  | 0.094 | **0.031** | 0.507 |
| ACD |  |  |  |  |  |  | 0.038 | -0.037 |
|  |  |  |  |  |  |  | 0.752 | 0.783 |
| hTERT |  |  |  |  |  |  |  | -0.004 |
|  |  |  |  |  |  |  |  | 0.9768 |

Supplementary table 6: Representation of ‘r’ and ‘p’ values by spearman correlation between mRNA levels of Shelterin complex genes, hTERT, mTL and clinical parameters.

| **r** | **TRF1** | **TRF2** | **POT1** | **RAP1** | **TIN2** | **ACD** | **hTERT** | **mTL** |
| --- | --- | --- | --- | --- | --- | --- | --- | --- |
| **p** |  |  |  |  |  |  |  |  |
| Total Protein | 0.197  0.095 | 0.118  0.323 | 0.037  0.755 | 0.198  0.095 | -0.036  0.758 | -0.025  0.833 | 0.008  0.945 | 0.125  0.383 |
| Albumin | -0.315  **0.0069** | -0.108  0.3659 | 0.189  0.110 | -0.087  0.467 | 0.058  0.623 | -0.030  0.799 | -0.027  0.818 | -0.152  0.292 |
| Globulin | 0.214  0.699 | 0.198  0.1084 | 0.001  0.991 | 0.232  **0.049** | -0.055  0.642 | 0.019  0.872 | -0.109  0.361 | 0.112  0.437 |
| Bilirubin total | -0.163  0.169 | -0.049  0.679 | 0.001  0.989 | -0.119  0.316 | -0.107  0.369 | 0.114  0.340 | -0.186  0.116 | -0.093  0.516 |
| Bilirubin direct | -0.245  **0.037** | -0.055  0.644 | -0.101  0.398 | -0.158  0.182 | -0.092  0.437 | -0.195  0.105 | -0.167  0.159 | -0.007  0.956 |
| Bilirubin indirect | -0.043  0.714 | 0.126  0.289 | 0.120  0.312 | -0.259  **0.028** | -0.109  0.359 | 0.056  0.639 | -0.121  0.311 | -0.075  0.603 |
| SGOT | -0.021  0.859 | -0.005  0.960 | 0.041  0.729 | 0.059  0.620 | -0.087  0.467 | 0.182  0.127 | -0.057  0.633 | 0.133  0.354 |
| SGPT | 0.007  0.946 | -0.010  0.931 | 0.018  0.879 | -0.104  0.383 | 0.067  0.572 | 0.093  0.440 | -0.107  0.368 | 0.085  0.553 |
| Calcium | 0.100  0.402 | 0.105  0.375 | 0.039  0.740 | 0.023  0.845 | -0.049  0.681 | 0.270  **0.022** | -0.051  0.665 | -0.015  0.912 |
| ALP | -0.192  0.106 | -0.119  0.318 | -0.238  **0.043** | -0.054  0.651 | 0.147  0.215 | -0.108  0.369 | -0.106  0.374 | 0.044  0.760 |
| Sodium | -0.082  0.489 | -0.180  0.129 | 0.042  0.724 | -0.143  0.228 | 0.291  **0.012** | -0.078  0.517 | 0.182  0.125 | 0.148  0.302 |
| Potassium | 0.124  0.299 | -0.044  0.711 | 0.093  0.434 | 0.067  0.573 | -0.095  0.423 | 0.199  0.096 | -0.069  0.561 | 0.098  0.496 |
| Blood urea | 0.044  0.709 | -0.010  0.931 | -0.113  0.344 | 0.128  0.283 | -0.194  0.102 | -0.008  0.944 | 0.146  0.220 | 0.037  0.797 |
| Uric acid | 0.118  0.321 | 0.134  0.261 | 0.064  0.592 | -0.007  0.953 | -0.040  0.734 | 0.1005  0.404 | -0.062  0.603 | 0.187  0.192 |
| Creatinine | 0.062  0.600 | -0.133  0.262 | -0.026  0.825 | 0.1531  0.199 | -0.259  **0.027** | -0.103  0.391 | -0.018  0.879 | 0.145  0.314 |
| Hb | -0.173  0.146 | -0.116  0.331 | 0.073  0.541 | 0.061  0.607 | 0.252  **0.032** | -0.007  0.951 | 0.079  0.508 | -0.088  0.539 |
| ESR | 0.239  **0.043** | -0.014  0.901 | -0.062  0.600 | 0.061  0.607 | 0.152  0.200 | -0.033  0.782 | 0.091  0.445 | 0.045  0.754 |

**Supplementary figures:**

Supplementary Figure 1: Box Whisker plot showing relative mRNA expression of A)TRF1, B)TIN2, C)ACD, D)hTERT and E) mTL.


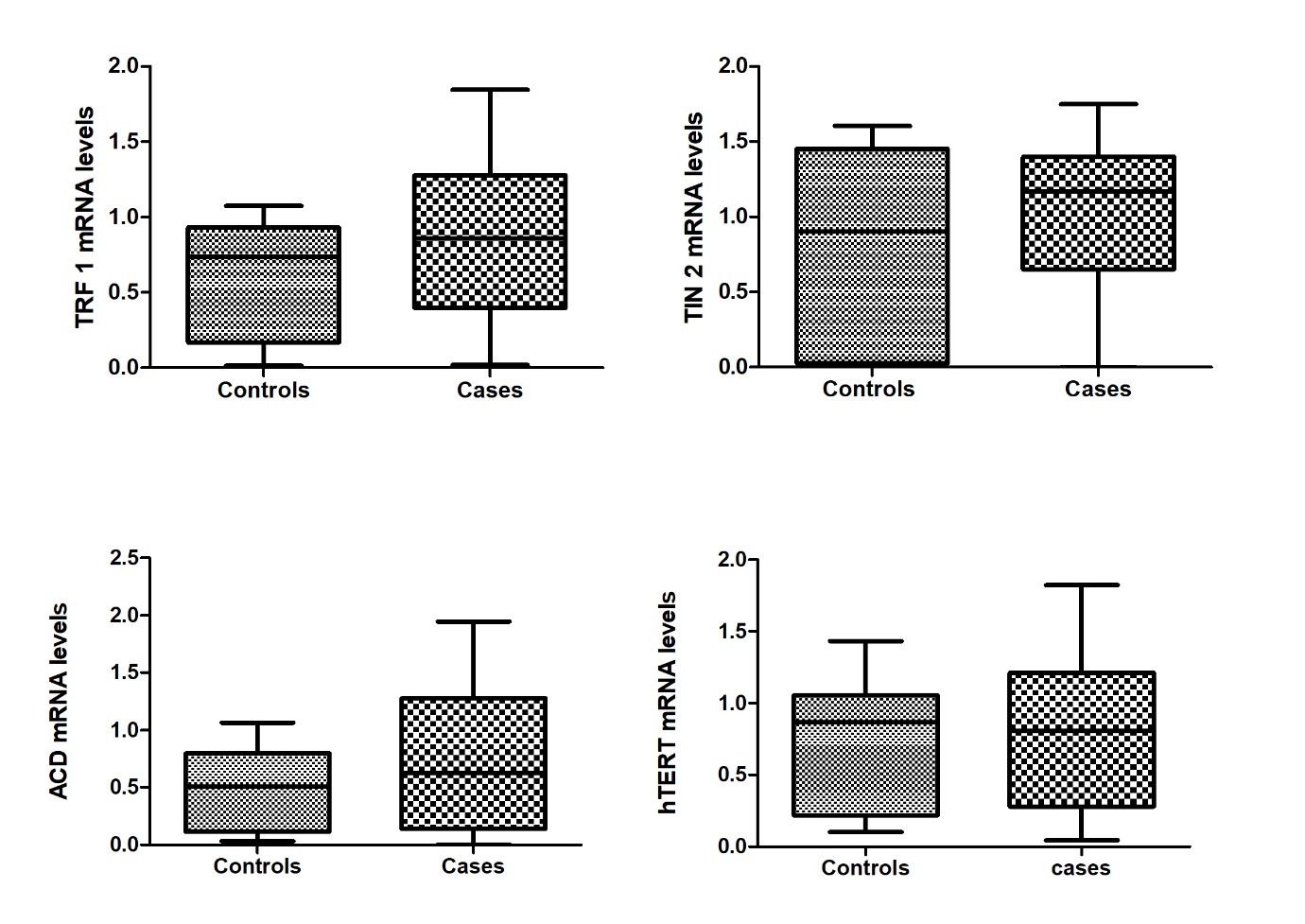


Supplementary Figure 2:: Kaplan Meier curve showing overall survival for MM patients stratified by A) TRF1 (P = 0.873), B) TRF2 (P = 0.929), C) POT1 (P = 0.943), D) TIN2 (P = 0.421), E) ACD (P = 0.908) and F) mTL (P = 0.304).


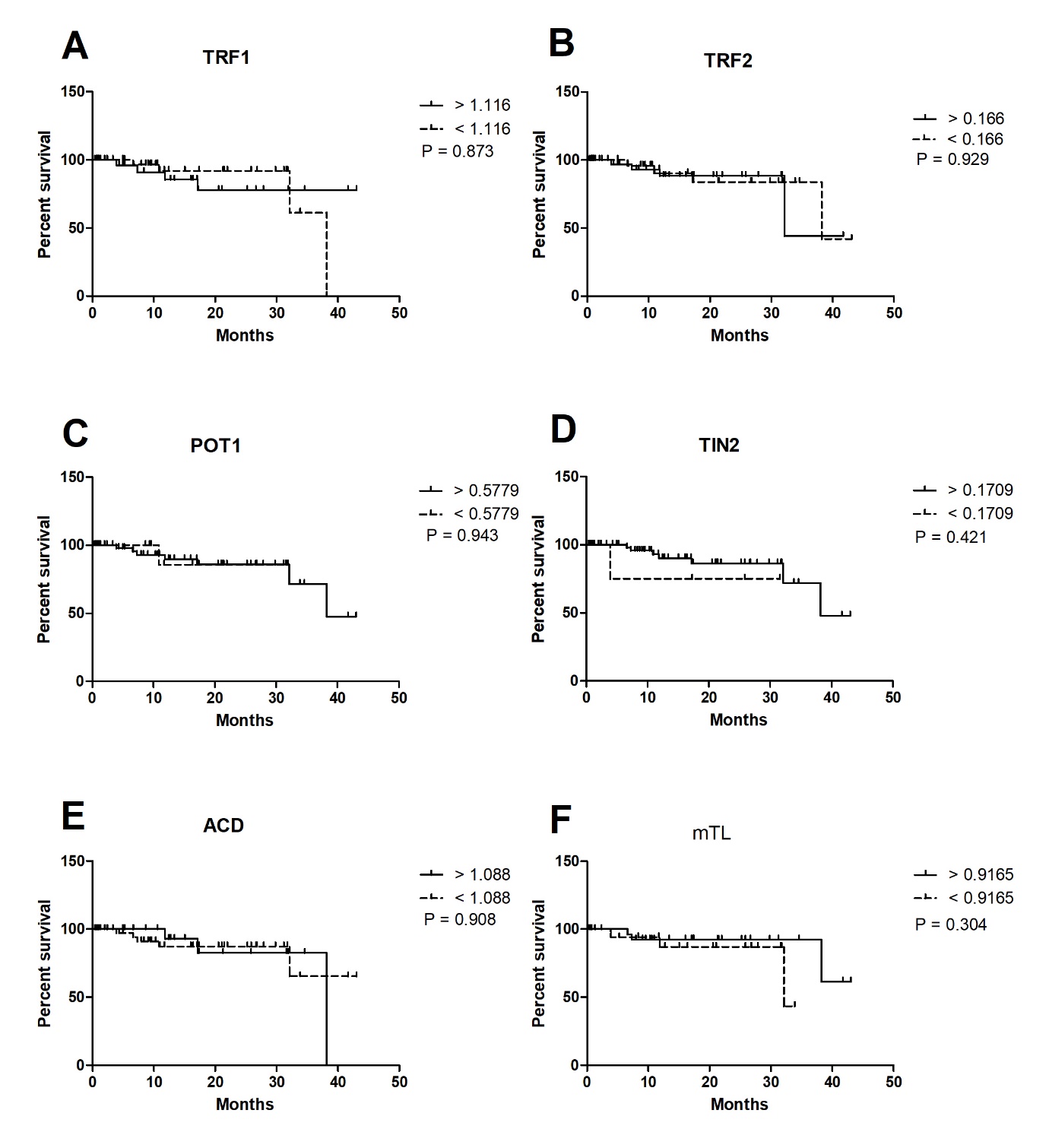

Supplement: Supplementary file 1 — Additional file 1: Supplementary Table 1. Chromosomal abnormalities of the MM patients. Supplementary Table 2. Representation of mRNA expression levels of Shelterin complex genes, hTERT and mTL. Data represented in median (range). Supplementary Table 3. Representation of AUC (Area under Curve) obtained by Receiver operative curves. Supplementary Table 4. Kaplan- Meier analysis of all the genes. Supplementary Table 5. Representation of ‘r’ and ‘p’ values by spearman correlation between mRNA levels of Shelterin complex genes, hTERT and mTL. Supplementary Table 6. Representation of ‘r’ and ‘p’ values by spearman correlation between mRNA levels of Shelterin complex genes, hTERT, mTL and clinical parameters. Supplementary Figure 1. Box Whisker plot showing relative mRNA expression of A)TRF1, B)TIN2, C)ACD, D)hTERT and E) mTL. Supplementary Figure 2. Kaplan Meier curve showing overall survival for MM patients stratified by A) TRF1 (P = 0.873), B) TRF2 (P = 0.929), C) POT1 (P = 0.943), D) TIN2 (P = 0.421), E) ACD (P = 0.908) and F) mTL (P = 0.304). [file 43141_2023_504_MOESM1_ESM.docx]
